# Medium and long-term follow-up after ST-segment elevation myocardial infarction in a sub-Saharan Africa population: a prospective cohort study

**DOI:** 10.1186/s12872-019-1043-1

**Published:** 2019-03-20

**Authors:** Hermann Yao, Arnaud Ekou, Aurore Hadéou, Jean-Jacques N’Djessan, Isabelle Kouamé, Roland N’Guetta

**Affiliations:** Intensive Care Unit, Abidjan Heart Institute, 01 BPV 206 Abidjan, Abidjan, Côte d’Ivoire

**Keywords:** ST-segment elevation acute myocardial infarction, Mortality, Sub-Saharan Africa

## Abstract

**Background:**

Major in-hospital mortality rate in patients with ST-segment Elevation Myocardial Infarction (STEMI) in Sub-Saharan Africa has been reported. Data on follow-up in these patients with STEMI are scarce. We aimed to assess medium and long-term prognosis in patients with STEMI admitted to Abidjan Heart Institute.

**Methods:**

Prospective cohort study including 260 patients admitted for STEMI to Abidjan Heart Institute, from January 1, 2012 to December 31, 2015. We compared mortality and nonfatal cardiovascular complications in revascularized and non-revascularized groups. Survival curve was generated with the Kaplan-Meier method. Predictors of mortality after STEMI were determined by multivariable Cox regression.

**Results:**

Of the 260 patients followed up on a median period of 39 months [28–68 months], 94 patients (36.1%) were revascularized and 166 (63.8%) were non-revascularized. Crude all-cause mortality was 10.4%. It was significantly higher in non-revascularized patients (*p* = 0.04). There was no difference in the occurrence of nonfatal cardiovascular complications in the 2 groups. In multivariable Cox regression, age ≥ 70 years, female gender and heart failure were the predictive factors for death after adjustment.

**Conclusions:**

STEMI remains an important cause of mortality in our practice. Healthcare policies should be developed to improve patient care and long-term outcomes.

## Background

Accounting for 15.2 million deaths in 2016, cardiovascular diseases are the leading causes of death worldwide [1]. Coronary heart disease (CHD) remains the major cause of mortality, with 19.0% of all-deaths [2]. In low and middle-income countries, mortality rate of CHD has steadily risen past years, and now is exceeding the burden of infectious diseases and other non-communicable diseases [1].

Major reduction of mortality rate in ST-segment Elevation Myocardial Infarction (STEMI) has been reported in developed countries [3, 4]. This is related to early reperfusion therapy and practice guidelines derived from observational registries [5, 6]. Data from FAST-MI 2015 registry [3] reported that STEMI mortality rate has declined markedly, from 10.2% in 1995 to 2.1% in 2015. After myocardial infarction, benefits of myocardial revascularization on short and long term outcomes has been largely described in Western countries [7].

In Sub-Saharan Africa (SSA), the increasing burden of acute coronary syndromes (ACS) and particularly STEMI is recognized past years [8–11]. Although high in-hospital mortality has already been documented by some studies, data on medium and long-term follow up of STEMI patients are scarce [9].

We therefore assessed the medium and long term mortality of STEMI patients in a SSA population.

## Methods

### Study design and population

This was a prospective, single centre study carried out between January 1, 2012 and December 31, 2015 and conducted in Intensive Care Unit of Abidjan Heart Institute, national referral center for the management of cardiovascular diseases in Côte d’Ivoire.

All consecutive adult (≥ 18 years of age) patients with diagnosis of STEMI, admitted to Intensive Care Unit of Abidjan Heart Institute, and alive at discharge, were included in the study. STEMI was confirmed if they had elevated serum markers of myocardial necrosis than the upper limit of normal for troponin and creatine kinase-MB; if they had symptoms of myocardial ischemia; and ECG changes on 2 contiguous leads with persisting ST-segment elevation ≥1 mm or pathological Q-waves or new onset of left bundle-branch block. Consent was obtained from each patient. Patients were followed-up until August, 2017 allowing us to have minimum follow-up period more than 18 months for each patient. The first day after hospitalization was considered as the beginning of the follow-up.

Patients were divided into two groups according to management: revascularized patients (thrombolysis alone, primary percutaneous coronary intervention (PCI), delayed PCI following or not thrombolysis) and coronary artery bypass graft surgery (CABG)) and non-revascularized patients.

Patients who died during hospitalization, refused to participate in the study, had unusable medical records, and were not reachable by telephone call (patient or family members) were not included in our study.

### End points

The outcomes variables used in the present study were:

- Primary end points: cardiovascular mortality and all-cause death.
- Secondary end points: recurrent non-fatal acute myocardial infarction (AMI), heart failure, sustained ventricular tachycardia, stroke.

### Data collection

Baseline data were entered into a standardized questionnaire during hospitalization. End points were obtained in medical records during follow-up visits. Data were collected via telephone calls to the patient or family members for patients who did not attend the follow-up appointments. We collected for each patient:

- Epidemiological data (age, sex, support mode)
- Clinical data (cardiovascular risk factors, clinical presentation)
- ECG, echocardiographic and laboratory findings
- In-hospital complications
- Mortality risk factor assessment: by GRACE score [12]
- Outcomes variables: primary and secondary end points.

### Statistical analysis

Continuous variables are presented as means ± standard deviation (m ± SD) or median [interquartile range]. Categorical data are presented as proportions. Statistical comparisons between groups used analysis of variance or Mann Whitney test for continuous variables and Chi-square test or Fisher exact test for categorical variables. We used Epi Info 3.5.8 (CDC, Atlanta, USA).

Survival curves were generated with the Kaplan-Meier method and compared by use of log-rank tests.

A backward stepwise Cox multivariable regression was used for assessing predictors of mortality, with a value of *p* <  0.25 for inclusion. The candidate variables included were selected on the potential to be associated with long term mortality in the literature: age, sex, history of AMI, hypertension, diabetes mellitus, active smoking, congestive heart failure at admission, left ventricular ejection fraction (LVEF), and revascularization. We defined statistical significance using a two-sided *p*-value < 0.05.

## Results

Of 329 patients with STEMI meeting our criteria, 47 died during hospitalization (14.3%); 282 patients were alive at discharge, and were eligible to participate to this study. Patients were divided into 98 revascularized (10 thrombolysis, 17 primary PCI, 69 delayed PCI (including 22 with previous thrombolysis), 2 CABG) and 184 with no reperfusion therapy.

The baseline characteristics of the patients are reported in Table 1. The median age was 57 years [48–64 years]. Most participants were men (sex ratio men/women = 4.7), statistically significant in revascularization group (*p* = 0.002). Patients didn’t have health insurance in the majority of cases.

**Table 1:** Baseline characteristics of the whole study population

Baseline characteristics	Total *N* = 282	Revascularization *n* = 98	No revascularization *n* = 184	*P*
Age (years), m [IQR]	57.0 [48.0–64.0]	55.0 [47.0–61.0]	57.0 [48.5–64.0]	0.11
Male sex	233 (82.6)	90 (91.8)	143 (77.7)	0.002
Health insurance	48 (17.0)	23 (23.5)	25 (13.6)	0.04
Hypertension	156 (55.3)	50 (51.0)	106 (57.6)	0.29
Diabetes mellitus	76 (27.0)	21 (21.4)	55 (29.9)	0.13
Active smoking	83 (29.4)	42 (42.9)	41 (22.3)	< 0.001
Dyslipidemia	101 (35.8)	41 (41.8)	60 (32.6)	0.12
Obesity	65 (23.0)	20 (20.4)	45 (21.8)	0.44
Physical inactivity	87 (30.9)	20 (20.4)	67 (26.3)	0.005
Prior MI	16 (5.7)	9 (9.2)	7 (3.8)	0.06
Killip stade ≥2	86 (30.5)	28 (28.6)	58 (31.5)	0.60
Heart rate (bpm), m [IQR]	84.0 [74.0–96.0]	81.5 [71.0–92.0]	87.0 [75.0–100.0]	0.03
Anterior wall MI	140 (49.6)	50 (51.0)	90 (48.9)	0.74
Arrythmias	60 (21.3)	29 (29.6)	31 (16.8)	0.01
Conduction disturmbances	68 (24.1)	21 (21.4)	47 (25.5)	0.44
LVEF(%), m [IQR]	51.0 [41.0–63.0]	48.0 [40.0–63.0]	53.0 [42.0–63.0]	0.27

Data are in n(%), otherwise they are specified. m [IQR]: median [interquartiles range]

*MI* Myocardial Infarction, *LVEF* left ventricular ejection fraction

The median delay onset of symptoms – admission to ICU was 20 h [6–72 h]. Patients who underwent early myocardial reperfusion procedure (primary PCI or thrombolysis) were admitted 5 h [2–6 h] after symptoms onset.

The proportion of patients presenting with heart failure (Killip class ≥2) was 30.5% (*p* = 0.60). Patients with no reperfusion therapy presented with higher heart rate (*p* = 0.03) and arrhythmias (*p* = 0.01).

In-hospital main complications were arrhythmias (27.0%) and heart failure (25.5%). Heart failure (*p* = 0.04) and extension of MI (12.5%, *p* = 0.04) often occurred in patients with no revascularization procedure. Mortality risk assessment by GRACE score showed a higher risk of death in non revascularized patients at 6 months (*p* = 0.02), 1 year (*p* = 0.04) and 3 years (*p* = 0.04) (Table 2).

**Table 2:** In-hospital non-fatal complications and mortality-risk assessment

Complications	Total *N* = 282	Revascularization *n* = 94	No revascularization *n* = 166	*P*
Arrythmias	76 (27.0)	30 (30.7)	46 (25.0)	0.31
Conduction disturmbances	44 (15.6)	13 (13.3)	31 (16.8)	0.62
Heart failure	72 (25.5)	18 (18.4)	54 (29.3)	0.04
Cardiogenic shock	13 (4.6)	4 (4.1)	9 (4.9)	0.99
Extension of MI	28 (9.9)	5 (5.1)	23 (12.5)	0.04
Thromboembolism	17 (6.0)	6 (6.1)	11 (6.0)	0.96
GRACE Score, m [IQR]	107 [86–126]	94 [85–120]	108 [88.5–127.0]	0.16
6 months MRA(%), m [IQR]	4.4 [2.6–7.9]	3.5 [1.6–6.2]	5.3 [2.6–8.8]	0.02
One-year MRA(%), m [IQR]	5.3 [2.7–11.0]	3.5 [2.1–6.9]	5.3 [2.9–11.0]	0.04
Three-years MRA(%), m [IQR]	11.0 [5.5–20.0]	8.6 [4.8–11.0]	11.5 [5.7–21.0]	0.04

Data are in n(%). m [IQR]: median [interquartiles range]

*MI* Myocardial Infarction, *MRA* mortality risk assessment by GRACE Score

**Fig. 1:**
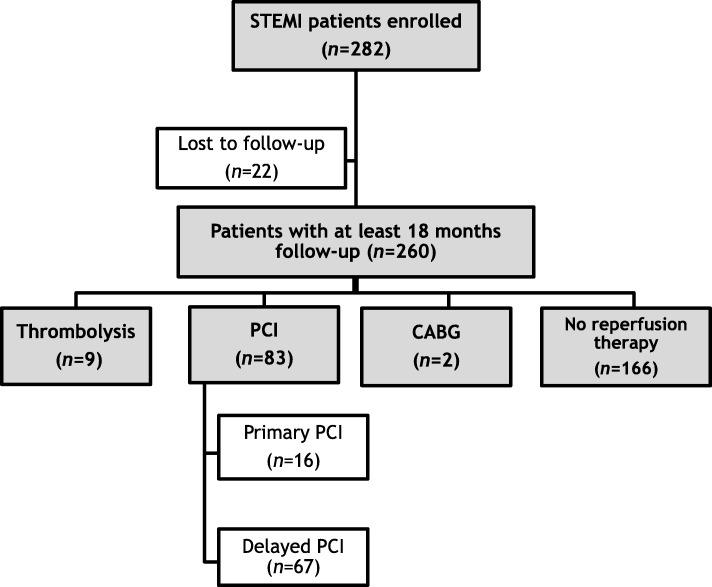
Study flow chart. STEMI: ST-segment Elevation Myocardial Infarction. PCI: Percutaneous Coronary Intervention. CABG: Coronary Artery Bypass Graft. Lost to follow-up: thrombolysis 1 patient, PCI 3 patients (primary PCI 1 patient, delayed PCI 2 patients), no revascularization 18 patients

After hospital discharge, follow-up was conducted on a median delay of 39 months [28–68 months]; 22 patients were lost to follow-up and information was obtained in 260 patients (Fig. 1).

**Fig. 2:**
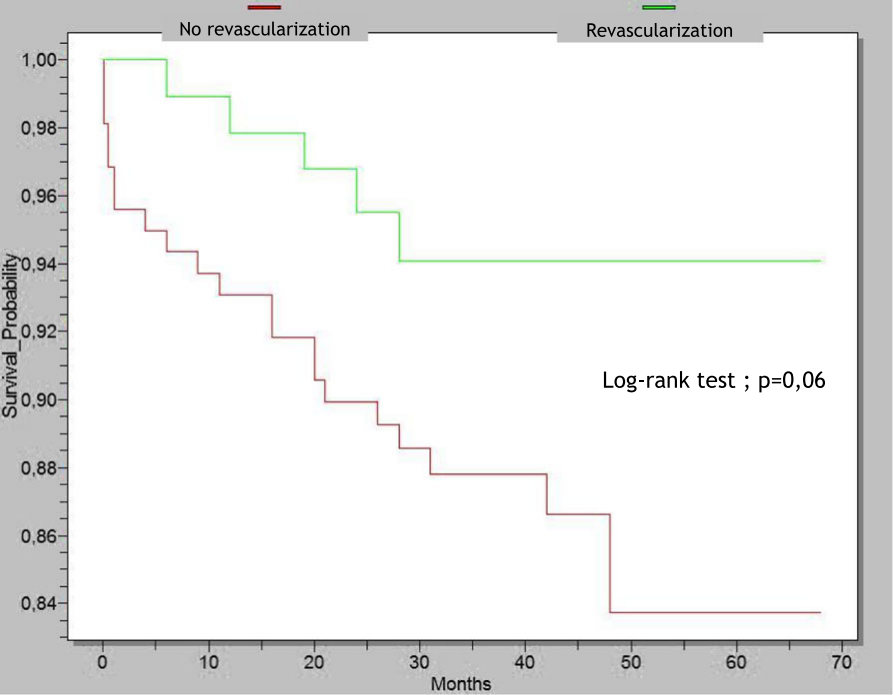
Survival curves according to in-hospital management

The overall mortality rate was 10.4%, and comparison between groups showed a significant higher mortality rate in no reperfusion therapy group (13.3 and 5.3% respectively, *p* = 0.04, HR 1.32, 95% CI 1.07–1.62) (Table 3). Survival curves generated by Kaplan-Meier method revealed a non-significant trend between the two groups (*p* = 0.06) (Fig. 2).

**Table 3:** Main complications during follow-up

Main complications	Total *N* = 260	Revascularization *n* = 94	No revascularization *n* = 166	*P*
Ventricular arrythmia	1 (0.4)	1 (1.1)	0 (0)	0.36
Heart failure	13 (5.0)	4 (4.3)	9 (5.4)	0.77
Myocardial infarction	11 (4.2)	4 (4.3)	5 (3.0)	0.72
Stroke	5 (1.9)	2 (2.1)	3 (1.8)	0.77
All-causes death	27 (10.4)	5 (5.3)	22 (13.3)	0.04

Data are in n(%)

In multivariable backward stepwise Cox analysis, age ≥ 70 years (HR 4,61, 95% CI 2.09–10.17), *p* <  0.001), female sex (HR 2.55, 95% CI 1.13–5.74, *p* = 0.02) and heart failure at admission (HR 2.17, 95% CI 1.01–4.69, *p* = 0.04) were associated with death after adjustment for history of MI, hypertension, diabetes mellitus, active smoking, LVEF and myocardial revascularization (Table 4).

**Table 4:** Predictors of long term all-cause death in multivariable Cox regression model

Predictors	Initial model			Final model		
	HR	95% CI	*P*	HR	95% CI	*P*
Age			0.002			< 0.001
< 70 years	1	–		1	–	
≥ 70 years	3.79	1.60–9.00		4.61	2.09–10.17	
Sex			0.07			0.02
Male	1	–		1	–	
Female	2.39	0.95–6.02		2.55	1.13–5.74	
Active smoking			0.51			
No	1	–				
Yes	0.69	0.22–2.14				
Heart failure			0.02			0.04
No	1	–		1	–	
Yes	2.58	1.14–5.83		2.17	1.01–4.69	
LVEF < 50%			0.06			
No	1	–				
Yes	2.21	0.96–5.13				
Revascularization			0.38			
Yes	1	–				
No	0.62	0.22–1.80				

*LVEF*: Left ventricular ejection fraction

## Discussion

Few data reporting short and long terms outcomes after STEMI are available in SSA [10, 11]. This evidence is related to paucity of studies on ACS in published articles [12]. However, SSA has experienced recent years a sharp rise of ACS [8–10]. To the best of our knowledge, this is the first real-world study in SSA comparing long-term mortality rate in STEMI patients, regarding in-hospital management, and after 39 months median delay period [28–68 months]. In a previous study, The ACCESS registry – South Africa reported 30 days and one-year outcomes after ACS [10]. Thirty-day and one-year death rates were respectively 2.4 and 6.7% [10]. Our study reported a higher overall death rate in patients with STEMI (10.4%). It seemed to be underestimated, because of patients lost to follow-up and potential selection bias. Many STEMI patients probably died before admission, or were admitted to other hospitals. These factors may explain a higher incidence of STEMI and thus a higher real-world mortality rate.

In SSA, in-hospital mortality rate (11.3–21%) [8–11] and long-term mortality rate are relatively high, due to rising of CHD, driven by changes in lifestyle and increased prevalence of cardiovascular risk factors [13, 14]. Prolonged treatment delays and limited healthcare facilities with reperfusion therapy have been clearly identified as factors affecting the outcomes in STEMI patients in SSA [15]. Current European guidelines emphasized importance of STEMI diagnostic as soon as possible and primary PCI as the preferred reperfusion strategy [5]. In SSA, extreme scarcity of catheterization laboratories and lack of interventional cardiologists results in variable rate of PCI in STEMI patients across countries [10, 16]. In our study, PCI was performed in 30.5% of cases, with primary PCI in 19.8% (17/86). In Kenya, primary PCI was performed at a similar lower rate (13%) [17]. In South Africa [10], 59.7% of STEMI patients underwent PCI. In wealthy countries, primary PCI rate is very high, between 52.7 and 71% [3, 4].

Early fibrinolytic treatment or pharmaco-invasive strategy appears as a good alternative therapy in SSA. Thrombolysis was performed in 32/282 STEMI patients (11.3%), 10.2% in Senegal [11] and 18% in South Africa [10]. Pharmaco-invasive strategy was used in majority of cases (22/32). Recently, the STREAM trial [18] revealed similar benefits in all-cause mortality between primary PCI and early pharmaco-invasive strategy. The Comparison of Angioplasty and Prehospital Thrombolysis in Acute Myocardial Infarction (CAPTIM) trial, has suggested that prehospital fibrinolytic therapy followed by PCI could do at least as well as primary PCI up to 5 years after STEMI [19]. A lower mortality has been reported in patients who underwent fibrinolytic treatment within 90 min or 2 h after symptoms onset [7, 19]. Overall, in the absence of contraindication and considering guidelines, treatment delay and limited access to interventional cardiology, a pharmaco-invasive strategy seems to represent a safe alternative to primary PCI [5].

Myocardial revascularization alone was not associated with lower death rate in STEMI patients in our study. In Western countries, it is early reperfusion therapy with primary PCI or fibrinolytic treatment that significantly reduced mortality after STEMI [7]. Median delay onset of symptoms – admission was 20 h [6 h - 72 h], still high but steadily decreasing since a recent study on ACS in Abidjan (44.7 h) [8]. This excessive delay influence reperfusion therapy rate in acute phase. Delay to treatment is a major contributor to mortality associated with STEMI [20]. Healthcare policies should focus on strategies in order to reduce this delay.

Age ≥ 70 years, heart failure and female sex were associated risk factors for long-term mortality in STEMI patients. Elderly and heart failure were already reported in multinational ACCESS registry, including 3 countries in North Africa and South Africa [10, 21]. In developed countries, previous studies revealed female sex as a risk factor for short-term [22] and long-term [23] mortality after STEMI. After adjustment for age, coexisting comorbidities and medications, difference in mortality rate between men and women seemed to be similar [22, 23]. These specific groups should have sustained care and secondary prevention.

Limited access to new therapy drugs also plays a key role in long-term mortality after STEMI, particularly new P2Y_12_ inhibitors (ticagrelor and prasugrel). These drugs have a greater potency, and are superior to clopidogrel in clinical outcomes in reducing mortality and major adverse cardiovascular events [24, 25]. These antiplatelet agents are not available in our practice. Nevertheless, all post-MI drugs (beta-blocker, aspirin, statin and renin-angiotensin system inhibitor) are important and yielded a benefit in cardiovascular mortality. Non-adherence to these medications increased 1-year mortality almost 4-fold [26]. In a previous study including ACS patients in SSA, only 80.2% of patients continued to take aspirin, 53.8% beta-blockers and less than 50% renin-angiotensin system inhibitors at 1 year [10].

Clinical studies have shown a beneficial effect of cardiovascular rehabilitation (CR) in patients with AMI [27]. In a recent study in France [27], CR decreased the risk of death at 5 years by 24%, after adjustment for sex, age, treatment at discharge, LVEF and revascularization procedures. In SSA, CR is yet incipient [28] and next years, should help improve patient survival, and quality of life after STEMI.

Face with these short-comings, and given increasing incidence of CHD in SSA, optimizing the management of STEMI in SSA remains a challenging issue. African countries have limited access to healthcare facilities capable of providing appropriate acute care. European and North American guidelines are less applicable in African countries and must be adapted to African specificities. A consensus statement has been proposed in AFRICARDIO-2 conference by an expert committee [15]. Health care policies should be implemented, with selected and achievable objectives: education about ACS symptoms both in patients and first-line healthcare providers, identify first-line healthcare facilities which should be equipped with ECG, early thrombotic therapy with aspirin and clopidogrel after a diagnosis of ACS has been confirmed or strongly suspected, implementation of networks with cardiology referral centres and catheterization laboratories, and efficient emergency medical service (EMS) trained to pre-hospital fibrinolytic treatment [15].

### Limitation and strengthening

According to western registries, our study included very small numbers of patients and therefore, unpowered some statistical analysis. Patients participating to the study were consecutive STEMI patients admitted to Intensive Care Unit of Abidjan Heart Institute. This sample may not be representative of all patients in Abidjan with STEMI at the same period. This number is certainly underestimated considering high pre-hospital death rate (lack of education of patient about ACS symptoms, limited facilities, ineffective EMS …). However, our study was conducted in the national referral center for the management of ACS in Côte d’Ivoire, and potential selection bias may be attenuated. Information bias was observed, because of incomplete medical records, data obtained by telephonic call, and lost to follow-up. Nevertheless, this study reported real world data on follow-up in STEMI patients in our practice.

## Conclusion

In SSA, particularly in Côte d’Ivoire, STEMI remains an important cause of mortality. Healthcare development and financial support have to be encouraged, in order to enhance patient’s survival after STEMI. Furthermore, improvement in management of patients after STEMI requires implementation of ACS registries in African countries, to increase awareness of the ACS burden and develop strategies and practice guidelines adapted to our specificities. On a larger sample of STEMI patients, it would be interesting to assess others associated risk factors for mortality, and to establish benefits of early reperfusion therapy on long-term prognosis.

## Abbreviations

ACS: Acute coronary syndrome
AMI: Acute myocardial infarction
CABG: Coronary artery bypass graft
CHD: Coronary heart disease
CR: Cardiac rehabilitation
EMS: Emergency medical service
LVEF: Left ventricular ejection fraction
PCI: Percutaneous coronary intervention
SSA: Sub Saharan Africa
STEMI: ST-segment elevation myocardial infarction
